# Comparative Study of Traditional Single-Needle Electrospinning and Novel Spiral-Vane Electrospinning: Influence on the Properties of Poly(caprolactone)/Gelatin Nanofiber Membranes

**DOI:** 10.3389/fbioe.2022.847800

**Published:** 2022-03-18

**Authors:** Qi Xu, Wei Liu, Bingcheng Yi

**Affiliations:** ^1^ Plastic Surgery Research Institute, Weifang Medical University, Weifang, China; ^2^ Department of Plastic and Reconstructive Surgery, Shanghai Key Laboratory of Tissue Engineering, Shanghai Ninth People’s Hospital, Shanghai Jiao Tong University School of Medicine, Shanghai, China

**Keywords:** single-needle electrospinning, spiral-vane electrospinning, poly(caprolactone), gelatin, high-throughput production

## Abstract

Spiral-vane electrospinning (SVE), a novel needleless electrospinning, was proven effective in obtaining high-throughput production of nanofibers. However, the properties of the electrospun nanofibers produced by SVE remain relatively underexplored, especially in comparison with those made by traditional single-needle electrospinning (SNE). Hence, for the comparative study of SNE and SVE in this study, the difference in the preparation mechanism was first analyzed using numerical simulation, followed by the experimental analysis of the effects of spinneret types on the quality and biocompatibility of electrospun poly(caprolactone)/gelatin (PCL/Gel) nanofibers. The values predicted by the electric field results were consistent with the experimental data, showing that the PCL/Gel nanofibers prepared by SVE have higher yields than SNE. Although the different spinnerets (i.e., needle and spiral vane) had little effect on the surface chemistry, thermal stability, and composition of the PCL/Gel nanofibers, they had great effects on the fiber diameter distribution and mechanical properties in which SVE-electrospun nanofibers have the wider diameter distribution and higher softness. Furthermore, the SVE-electrospun nanofibers were also proven to exhibit good biocompatibility for cell growth of human adipose-derived stem cells (hADSCs) and cell–fiber interactions. Summarily, compared to the traditional SNE, SVE-electrospun nanofibers exhibited many merits including high-throughput yield, good air permeability, and compliance, which provide a facile and effective platform for the improvement of nanofiber applications in biomedical fields (e.g., tissue engineering, cosmetic, and medical textiles).

## Introduction

Owing to the characteristics of high specific surface area for biomolecule binding and biomimetic extracellular matrix (ECM)-like fibrous structure for cell growth, nanofibers are developed and widely used in the various biomedical fields, such as skin-beauty, wound dressings, and tissue engineering (Xue et al., 2019; Xie et al., 2020; Yi et al., 2021a; Wang et al., 2021). Among the current developed technologies for preparing nanofibers (e.g., phase separation (Kang et al., 2020), self-assembly (Wu et al., 2020), and electrospinning (Xue et al., 2019)), electrospinning is regarded as a viable and highly versatile method to massively prepare continuous ultrafine fibers from various polymers and composites. However, traditional electrospinning using a single needle to supply spinning solution lacks efficiency, resulting in the low productivity of the electrospun nanofibers for commercial application (Zhang et al., 2020). To meet the increased demand for nanofibers, remarkable progress has been made concerning the increase of the jet number for high-throughput production of nanofibers. For example, multi-needle electrospinning is developed as a straightforward and effective way to increase the electrospinning throughput (Zhu et al., 2021). Nevertheless, it has exhibited obvious shortcomings including complicated design and potential clogging of the spinneret that may lead to reduced fiber production rate and poor fiber quality (Liu and Guo, 2013), which seriously limits the wide application of this technique in the industry.

In this context, needleless electrospinning that initiates the charged jets from the open free-liquid surface is exploited and demonstrated to present some unique advantages compared to multi-needle electrospinning, especially in the achievement of nanofibers with high throughput (Yin et al., 2021). It has been confirmed that the free surface of the spinning solution is subjected to unsteadiness and begins to produce tiny fluctuations under the action of a high enough electric field during the needleless electrospinning process. Once the surface tension of each peak position of the tiny fluctuations is overcome, numerous jets are formed and stretched into nanofibers (Jiang et al., 2019). Thus, the jet initiation and resulting fiber morphology rely heavily on the electric field intensity profile around the spinneret and in the electrospinning zone that is governed by the applied voltage and the shape of the needleless spinneret (Niu et al., 2009). Recently, needleless electrospinning setups have been developed, in which spinnerets with various geometries, such as cylinder, disk, ball, and coil, were used to produce nanofibers (Jirsak and Petrik, 2012; Wang et al., 2012; Yu et al., 2017; Ranjbari et al., 2020). Under the same applied voltage, these spinnerets present fairly different electric field profiles. To reveal the influence of spinneret shape on the electric field, efforts have been made and demonstrated that spiral vane would be the most prominent spinneret of needleless electrospinning that concentrates the strongest electric field in the largest areas of itself (Wang et al., 2012).

Hence, as a newly exploited strategy of needleless electrospinning, spiral-vane electrospinning (SVE) was regarded as a very efficient device for preparing nanofibers with high quality and yield (Gao et al., 2016; Liu and Zuo, 2018; Liu and Zuo, 2020), and the yield of nanofibers is usually positively correlated with the size of the spiral vane. As reported by Gao et al. (2016), SVE was performed to fabricate nanomembranes with a certain thickness, aiming to improve their sound absorption properties. Later, Liu and Zuo (2018) and Liu and Zuo (2020) used SVE to attain nanofiber membranes with better sound absorption property by modifying polyvinyl alcohol nanofiber membranes. These findings establish significant supremacy for the aggressive promotion of nanofibers to be applied in the market. Nevertheless, in addition to the yield of nanofibers, little has been reported in the literature on how the nozzle structure influences the needleless electrospinning process and resulting fiber morphology in comparison to traditional single-needle electrospinning, which serves as an important indicator for the reference of SVE performances. Therefore, to benefit the development and application of needleless electrospinning in the industry, it is worthwhile to perform a comparative study of the novel spiral-vane electrospinning and the traditional single-needle electrospinning.

In tissue engineering, polymer blending for electrospinning is one of the most effective methods to provide desirable biocomposite nanofibers for the guidance of tissue remodeling (Ghasemi-Mobarakeh et al., 2008). For example, a synthetic polymer can be blended with a natural polymer to obtain a fibrous scaffold with good biocompatibility and improved mechanical or other physical/chemical properties. A typical example is the blending of poly(3-caprolactone) (PCL) lacking surface cell-recognition sites and gelatin (Gel) suffering from poor mechanical properties. Such composite fibers can overcome their shortcomings and produce a new biomaterial with desired cell adhesion and degradation rate (Zhang et al., 2005; Ghasemi-Mobarakeh et al., 2008). Thus, PCL/Gel nanofibrous scaffolds were used in this study for the comparative study of SNE and SVE. In brief, the electrospinning processes and mechanisms were first observed and analyzed between SNE and SVE. Then, the material properties of SVE-generated PCL/Gel nanofibers were compared to those of SNE-generated PCL/Gel nanofibers. Furthermore, the cellular responses of human adipose-derived stem cells (hADSCs) to two PCL/Gel nanofibers were studied to understand the difference in their biofunctions between SNE and SVE.

## Materials and Methods

### Fabrication of PCL/Gel Nanofibers

Using 1,1,1,3,3,3-hexafluoro-2-propanol (HFIP) as the dissolving solvent, the mixed polymer solution of PCL (M_w_ 550,000, Jinan Daigang Biomaterials) and Gel (type A, Shanghai Yeasen Biotechnology) with the weight ratio of 70:30 was prepared at the total concentration of 10% w/v and stirred for 12 h at room temperature. After the dissolved solution was transferred into a syringe with a 19 G blunt-tip needle, SNE was performed to generate PCL/Gel nanofibers using the following parameters: applied voltage 13 kV, flow rate 1 ml/h, needle tip to collector gap distance 15 cm, and ambient conditions (20–25°C, 25–30% humidity). According to the resulting fiber morphology relying heavily on the applied voltage and the shape of the needleless spinneret in needleless electrospinning (Jirsak and Petrik, 2012; Yu et al., 2017), the applied voltage was mainly regulated during the SVE process to form the nanofibrous scaffolds with a similar diameter to those formed by SNE. Thus, SVE was performed as follows: applied voltage 60 kV, spiral vane to collector gap distance 15 cm, and spiral vane speed (6 cm in diameter, 10 cm in length) 5 rpm. During SNE and SVE, the emitted jets were monitored by using a digital camera. After manufacturing, all the electrospun nanofibrous membranes were dried for a week under vacuum to remove residual solvent.

### Simulation of Electric Field Distribution

The two-dimensional (2D) electric fields of the single-needle system (i.e., SNE) and spiral-vane system (i.e., SVE) were simulated by the COMSOL Multiphysics software (COMSOL Inc., Sweden). The physical geometries of these electrospinning setups (e.g., electrode, collector) were established according to their practical dimensions and locations. Then, all the electric field strengths and distributions were visualized using the software.

### Characterization of PCL/Gel Nanofibrous Membranes

**Fiber morphology:** surface morphologies of electrospun PCL/Gel nanofibers were observed by using a scanning electron microscope (SEM, ZEISS Gemini 300, Germany) at an acceleration voltage of 0.02–30 kV. Before SEM imaging, all the fibrous mats were sputter-coated for 30 s with gold to increase conductivity. The distribution of fiber diameter (n > 100) was further analyzed directly from the obtained SEM images using the ImageJ software (NIH, Bethesda, Maryland, United States).

**Surface chemistry and crystallinity structure:** Fourier transform infrared (FTIR) spectroscopy of the electrospun PCL/Gel nanofibers was qualitatively analyzed by using an FTIR spectrometer (Thermo Fisher IN10, United States) over the wavenumber range of 4,000–600 cm^−1^ at a scanning resolution of 2 cm^−1^. The crystalline structure of the PCL/Gel nanofibers was determined by using an X-ray diffractometer (XRD, Rigaku, Japan) at the operating voltage of 40 kV and current of 200 mA, respectively. After background subtraction, the crystallinity was calculated from the fractional area of the crystalline peaks to the total area.

**Thermal stability**: thermogravimetric analysis (TG) of the electrospun PCL/Gel nanofibers was carried out using thermal analyzer apparatus (NETZSCH STA 449F5, Germany). The 3–7 mg samples were heated in a nitrogen atmosphere in the temperature range from 30 to 800°C at the heating rate of 20°C min^−1^. Derivative thermogravimetry (DTG) was calculated from the derivate of the TG curve, which represents the difference in the thermogravimetry ratio of weight loss measurement at heating.

**Tensile properties:** tensile properties of the electrospun PCL/Gel nanofibers were determined using a tabletop tensile tester (5542-C8609, INSTRON Instrument, United States) equipped with a 50 N load cell at ambient conditions. All samples were cut into a rectangle with 3 cm length and 1 cm width and fixed on the fixture of the biomechanics analyzer. Then, the stress–strain curves of the samples (n > 8) were recorded under stretching at a cross-head speed of 10 mm/min until fracture. The fibers’ mechanical parameters such as Young’s modulus, tensile strength, strain at break, and maximum load were further obtained from the stress–strain curve.

### Cellular Responses of hADSCs to the PCL/Gel Nanofibers

**Isolation of the primary hADSCs and cell culture:** human adipose-derived stem cells (hADSCs) were extracted from adipose tissue according to the Animal Care and Experiment Committee of Shanghai Ninth People’s Hospital, Shanghai Jiao Tong University School of Medicine. In brief, adipose tissue was immersed in 0.25% chloramphenicol solution for 15 min; after thoroughly rinsing in PBS solution three times, the tissue was cut into pieces and then shaken for digestion in type IV collagenase at 37°C for 1 h. The obtained cell suspension was finally centrifuged at 1,500 rpm for 5 min and then resuspended and cultured with mesenchymal stem cell medium (Shanghai Zhongqiao Xinzhou Biotechnology Co., Ltd, ScienCell, United States) containing 5% fetal calf serum, 1% growth supplement, and 1% penicillin/streptomycin solution.

**Cell morphology:** hADSCs were seeded onto the fibrous membranes in 24-well plates at a density of 5×10^4^ cells/well. After 3 days of culture, the cell morphology was stained with TRITC-labeled phalloidin (1:200 dilution in PBS, Yeasen Biotech Co., Ltd., Shanghai), and the cell nuclei were stained with 0.8 mg/ml 4′,6-diamidino-2-phenylindole (DAPI, SouthernBiotech, United States) for 10 min at room temperature in the dark. Morphology of the hADSCs was observed using a laser confocal scanning microscope (Carl Zeiss, Germany). ImageJ further analyzed cell numbers based on the obtained microscope images.

**Cell proliferation:** hADSCs were seeded onto the PCL/Gel nanofibers at a density of 1×10^4^ cells/well in 24-well plates. According to the manufacturer’s instruction, after 1, 3, 5, and 7 days of culture after seeding, cell proliferation was monitored by using the Cell Counting Kit-8 (CCK-8, Dojindo, Japan). In brief, the cellularized fibrous mats were incubated with a 300 μL culture medium containing 15 μL of CCK-8 at 37°C for 3.5 h. Then, 100 μL of the solution was transferred to a 96-well plate for absorbance measurements at 450 nm using a microplate reader (ARIOSKAN, Thermo Scientific, Germany) to analyze cell numbers.

**Cell spreading:** cells were seeded onto the PCL/Gel nanofibers at a density of 5×10^4^ cells/well in 24-well plates. After culturing the cell–fiber constructs for 2 days, cell spreading was observed by scanning electron microscopy (SEM, ZEISS Gemini 300, Germany). In brief, the samples were washed with PBS three times and fixed overnight at 4°C in 2.5% glutaraldehyde and then dehydrated through a graded series of ethanol (30–100%, v/v) at 4°C for 15 min at each step. Once dried, the samples were examined by SEM at an acceleration voltage of 0.02–30 kV.

**Live/dead staining:** cells were seeded onto the PCL/Gel nanofibers at a density of 5×10^4^ cells/well in 24-well plates. After culturing the cell–fiber constructs for 3 days, live/dead assays (Dojindo, Shanghai) were used to evaluate the viability of hADSCs. In brief, 0.2% (v/v) calcein-AM and 0.3% (v/v) propidium iodide were mixed into PBS to make a working solution. After 30 min of incubation with the working solution at 37°C, cell viability was observed under a fluorescence microscope (Axiocam 503 color, Carl Zeiss, Germany).

**Cell–fiber interaction:** hADSCs were seeded onto the PCL/Gel nanofibers in 24-well plates at a density of 5×10^4^ cells/well. After 3 days of culture, immunofluorescence staining was performed to examine the expression of integrin in cells to analyze the cell–fiber interaction. In brief, the cellularized fibrous mats were fixed using 4% paraformaldehyde for 30 min, permeabilized using 0.2% Triton X-100 for 5 min, and blocked using 10% goat serum for 30 min. After that, the rabbit anti-human integrin-β1 antibody (1:200 dilution, Proteintech, United States) was incubated overnight at 4°C, followed by the incubation of goat anti-rabbit IgG (H + L) secondary antibody (1:100 dilution, Proteintech, United States) for 90 min in the dark at room temperature. The cell nuclei were counter-stained with DAPI for 10 min at room temperature. Finally, the samples were observed using a laser confocal scanning microscope.

### Statistical Analysis

Data are presented as means ± standard deviation. Statistical analysis was carried out using the Origin 8.0 software, and Tukey’s HSD post hoc tests were used to make pair-wise comparisons between groups. The obtained *p* values (^∗^ *p* < 0.05, ^∗∗^ *p* < 0.01, and ^∗∗∗^ *p* < 0.001) were considered statistically significant. All of the experiments were repeated at least three times.

## Results and Discussion

### Wide Distribution of Electric Field in the SVE Process Brings in Nanofibers’ High-Throughput Production

**FIGURE 1:**
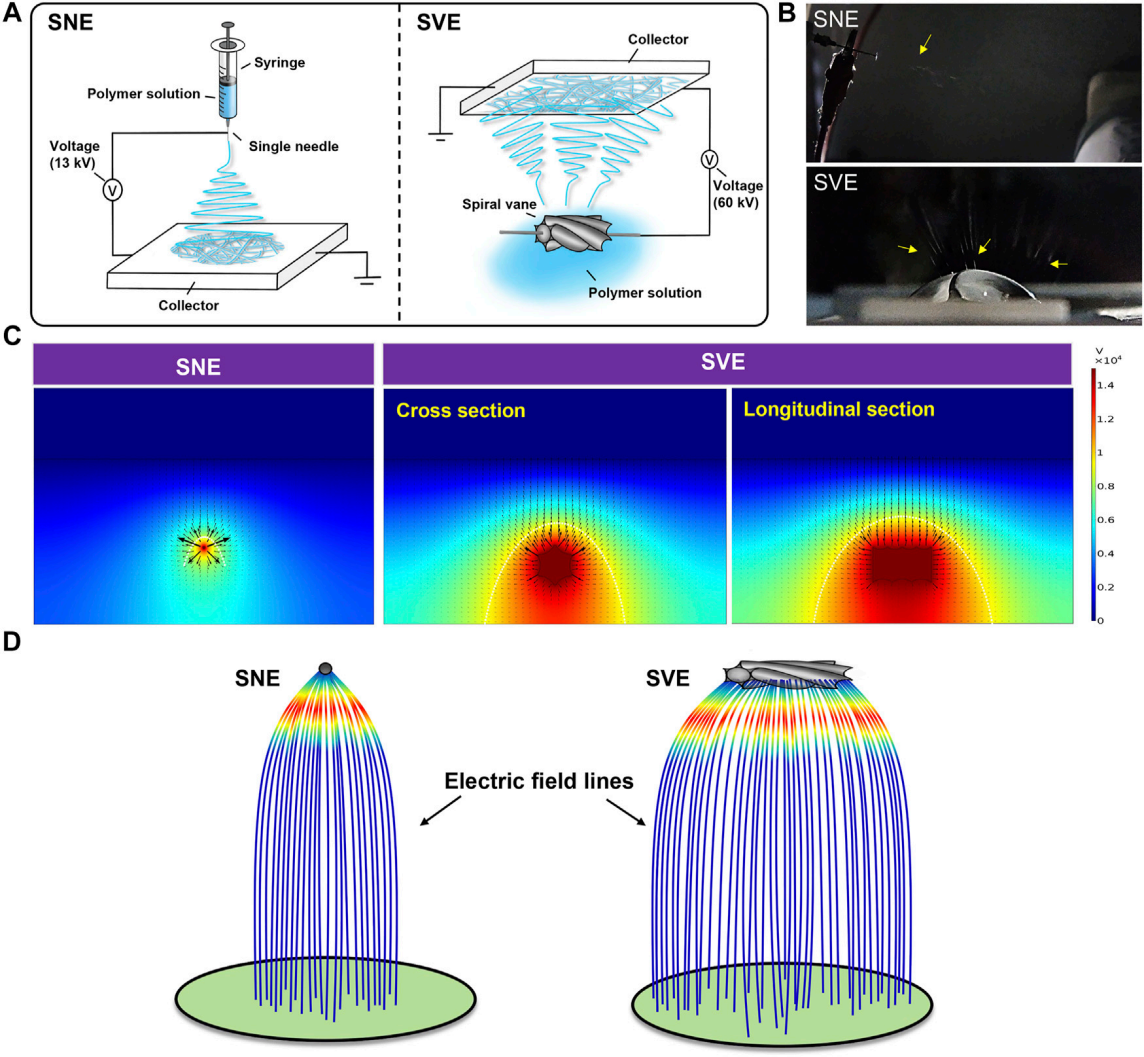
Mechanism analysis of SNE and SVE processes: **(A)** schematic diagrams; **(B)** pictures of electrospinning processes. The yellow arrows represent the generated jets. **(C)** Electric field distributions of single-needle and spiral-vane systems; **(D)** simulative electric field lines produced by the single-needle and spiral-vane spinnerets.

Spiral-vane electrostatic machine, using spiral vane as a free surface spinneret, is a current exploited strategy to achieve the high-throughput production of nanofibers among needleless electrospinning. Similar to the traditional SNE, SVE involves an electrohydrodynamic process. Each peak position of the tiny fluctuations was electrified to generate a jet under the action of a high enough electric field, followed by stretching and elongation into nanofibers (Figure 1A) (Xue et al., 2017). However, the charged precursor liquid was extruded from the needle spinneret to produce only one jet during traditional electrospinning resulting in the low yield of nanofibers (Figure 1B) (Yin et al., 2021). Moreover, to break the steady state of the solution surface for fluctuations and overcome the surface tension of the charged precursor solution for jet formation, higher voltage is usually required to be applied between the spiral vane spinneret end and the collector in SVE, whereas in the needle spinneret-based electrospinning, the applied electric voltage just needs to overcome the solution surface tension. For example, if the spiral vane spinneret was performed at a low applied voltage, the jets were only generated from two end areas, without any jets/filaments produced from the middle spiral vane surface. Further increasing the applied voltage can generate a high-intensity electric field and then lead to the generation of jets from the entire spiral vane surface, which is consistent with a previous study (Niu et al., 2009). Thus, to fabricate the PCL/Gel nanofibers with similar diameter in this study, the applied electric voltage of SVE was set as 60 kV, while that of SNE was 13 kV (Figure 1A).

Considering the critical role of the electric field in the electrospinning process, numerical simulation was further performed to visualize the electric field intensity and distribution. As illustrated in Figure 1C, the direction of arrows indicates the direction of the electric field, and the length of the arrows is proportional to the strength of the electric field at the corresponding position. The maximum *E*_z_ (z-directional electric field strength) at the needle spinneret and the spiral vane spinneret is different, in which the weak electric field is formed at the central position, while the strong electric field is at the outside position. Under this circumstance, the central jets suffer from a lower electrostatic force than the jets formed on the sides (Xie and Zeng, 2012). Furthermore, the bigger size of the spiral vane expands the electric field distribution in the region near the spinneret than that near the needle, creating more electrospinning area. As shown in Figure 1D, the electric field lines generated in the spiral vane spinneret are more likely scattered outward at the edge of the spinneret leading to scattered fiber mats, while those in the needle spinneret concentrated and intensified at the central position.

### SVE-Electrospun Nanofibers Exhibit Lower Crystallinity and Higher Compliance Than SNE-Electrospun Nanofibers

**FIGURE 2:**
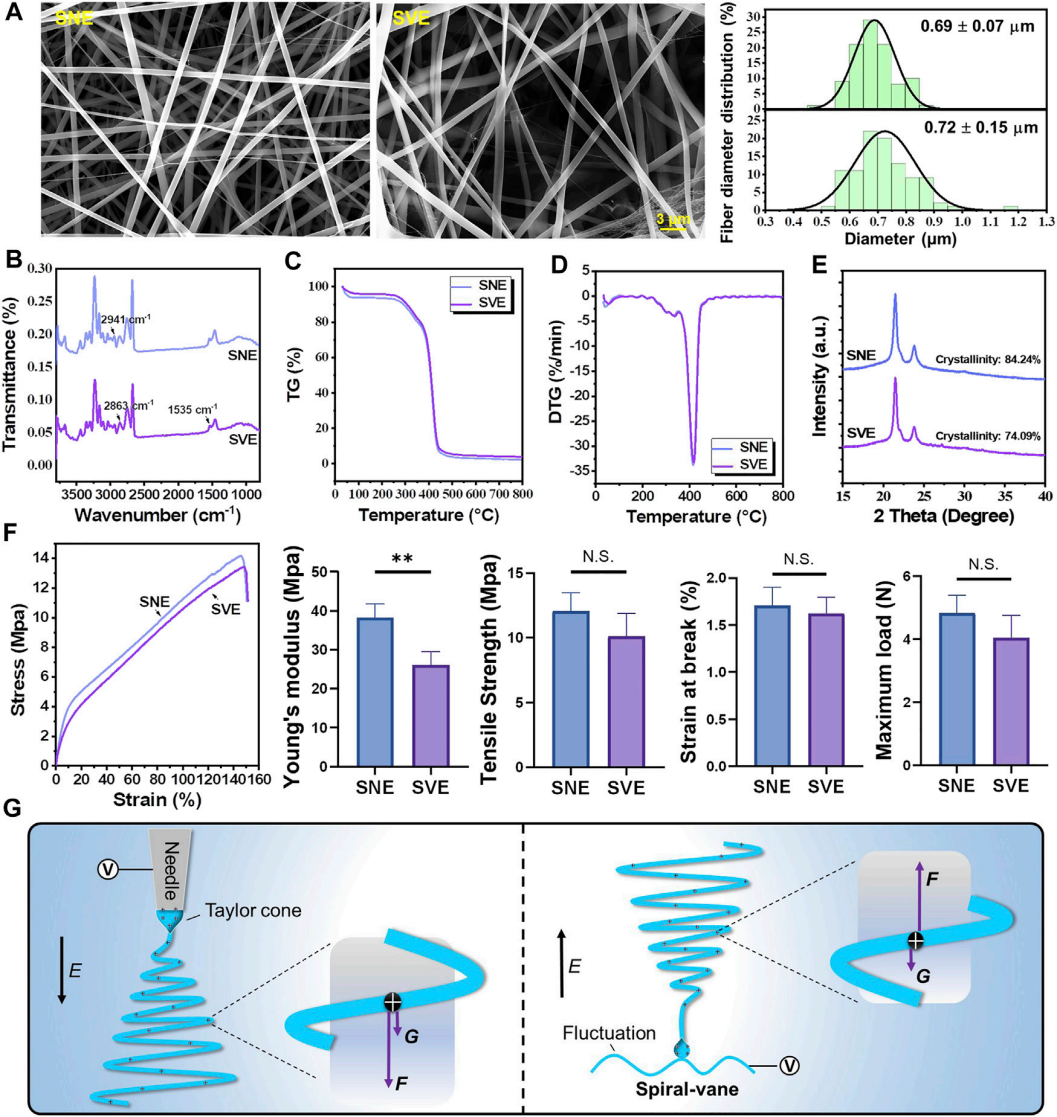
Characterization of the PCL/Gel nanofibers produced by SNE and SVE: **(A)** SEM and the distribution of fiber diameter; **(B)** FTIR; **(C,D)** thermogravimetry and derivative thermogravimetry; **(E)** XRD and crystallinity; **(F)** stress–strain curves, Young’s modulus, tensile strength, strain at break, and maximum load; and **(G)** the proposed mechanisms for molecule elongation and crystallinity. *E* indicates the direction of the electric field. *F* and *G* represent the electric force and gravity force actioned on jets, respectively.

Based on the PCL/Gel 70:30 formulation, the physical properties of electrospun nanofibers including fiber morphology, surface chemistry, thermostability, and mechanical properties were analyzed to perform the comparative study of SNE and SVE. SEM examination revealed the nonsignificant difference of average fiber diameter between SNE- and SVE-electrospun nanofibers (Figure 2A, *left*). However, the fiber diameter of SVE-electrospun nanofibers was observed to be distributed more widely than that of SNE-electrospun ones (0.69 ± 0.70 *vs*. 0.72 ± 0.15 μm, Figure 2A, *right*), which was also observed in other needleless electrospinnings (Yu et al., 2017). This mainly attributes to the larger and more nonuniform electric field generated near the spiral vane than near the needle, resulting in the significant difference of drawing force that existed in the jets for the production of nanofibers. Interestingly, the wide fiber diameter distribution in nanofibrous membranes is always accompanied by high porosity for excellent air permeability (Wanasekara et al., 2015).

FTIR analysis was carried out for the surface chemistry characterization of PCL/Gel nanofibers produced by SNE and SVE (Figure 2B). It can be seen that the FTIR spectra of SVE-electrospun nanofibers were nearly the same as those of SNE-electrospun nanofibers, in which infrared spectra for PCL-related stretching modes were observed at 2,941 cm^−1^ (asymmetric CH_2_ stretching) and 2,863 cm^−1^ (symmetric CH_2_ stretching) and those for Gel-related stretching modes appeared at approximately 1,535 cm^−1^ (a coupling of bending of the N-H bond and stretching of C-N bonds in amide II) (Ghasemi-Mobarakeh et al., 2008). Figures 2C,D show the thermal stability of SVE- and SNE-electrospun nanofibers. The obtained nearly similar TG and DTG curves further indicated that there was a nonsignificant difference in the thermal stability and composition of these electrospun PCL/Gel nanofibers. However, the crystallinity of SVE-electrospun PCL/Gel nanofibers was lower compared to that of SNE-electrospun nanofibers (Figure 2E, 74.09 vs. 84.24%). As the mechanical performance of nanofibers is well known to be closely connected with the molecular crystallinity, the mechanical properties of the electrospun PCL/Gel nanofibers were further examined. The stress–strain curve is shown in Figure 2F. It was found that the nanofibers from traditional SNE had Young’s modulus of 38.75 MPa, close to that reported by Zhang et al. (2005). In contrast, the mechanical properties of the SVE-electrospun nanofibers were significantly decreased with Young’s modulus of 25.86 MPa. The tendency is quite similar to that of molecular crystallinity. Nevertheless, the type of spinneret appeared to not significantly affect the ultimate tensile strength, the strain at break, and the maximum load of the PCL/Gel nanofibers (Figure 2F).

For the underlying mechanisms, it is well known that there are several forces (i.e., electrical forces, surface tension, inner viscoelastic force, and gravity force) exerting an influence on the fluid jets (Yu et al., 2011). Usually, surface tension and inner viscoelastic force are the inherent performances of the spinning solution which are not affected by the type of spinneret. This implies that the electrical and gravitational forces are mainly responsible for drawing and downsizing the fluid jets. As illustrated in Figure 2G, the action of the gravity force can be deduced to enhance the influence of electrical force on the stretching and elongation of polymer molecules during SNE, while that in the SVE process, in contrast, can weaken the stretched effect of the electrical force on the jets. The higher extensional forces stretching the polymer chains may consequently increase the molecular chain orientation by preventing the occurrence of molecule relaxation, in favor of enhancing crystallization in SNE-electrospun nanofibers (Yi et al., 2018).

### SVE-Electrospun PCL/Gel Nanofibers Exhibit Good Biocompatibility for Cell–Fiber Interaction

**FIGURE 3:**
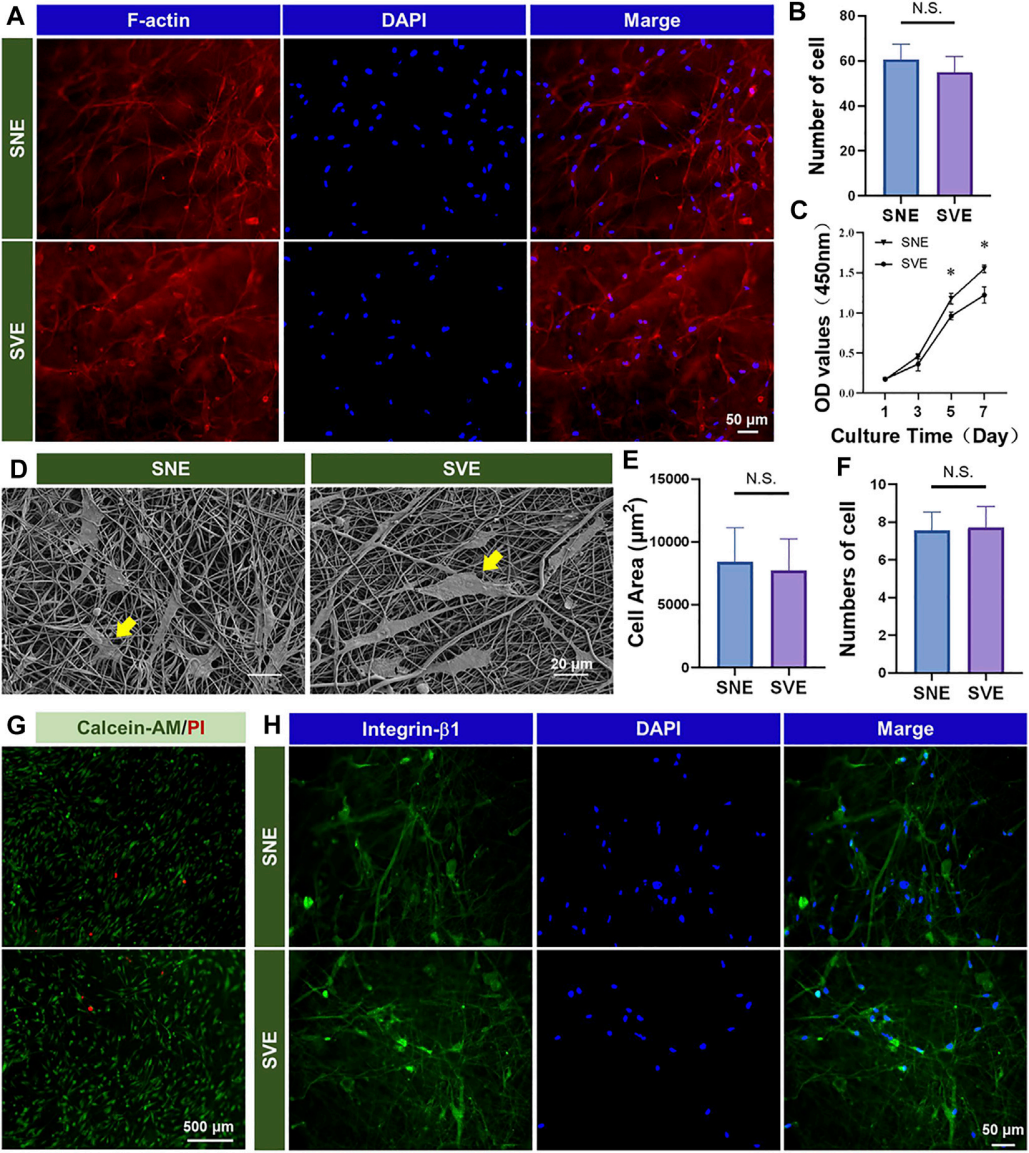
Biocompatibility of the PCL/Gel nanofibers produced by SNE and SVE: **(A,B)** morphology and quantified cell number of hADSCs on different samples; **(C)** cell proliferation; **(D–F)** cell spreading and quantified spreading area and adhered cell number on different samples; **(G)** cell viability; and **(H)** expression of integrin-β1 for cell–fiber interactions.

hADSCs were demonstrated to possess many of the same regenerative properties as mesenchymal stem cells (MSCs): the potential of multidirectional differentiation and the excellent ability to proliferate *in vitro* (Miana and Prieto González, 2018; Si et al., 2019). Hence, hADSCs were selected and chosen as the model cells to analyze the difference in the biocompatibility of the different PCL/Gel nanofibers. Cell morphology was first analyzed using a fluorescence microscope after 3 days of culture (Figure 3A). The results showed that both nanofibers appeared to support good cell growth and elongation. The quantified results on cell number indicated the subtle higher cell growth on SNE-electrospun nanofibers than SVE-electrospun nanofibers (Figure 3B). It is well known that when placed into scaffolds, cells can sense the physicochemical and biochemical properties (e.g., fiber stiffness, surface pattern, and surface chemistry) of the underlying substrates through cell membrane-bound receptors and then initiate the intracellular signaling cascades for the cellular responses including migration, proliferation, and differentiation (Fernandez-Yague et al., 2015). Due to no significant difference in the fiber diameter and surface chemistry of SNE- and SVE-electrospun PCL/Gel nanofibers, the increased modulus of nanofibers was deduced to play a key role in promoting cell growth (Yi et al., 2019). Likewise, a softer surface enhances cell secretory activity, while a stiffer substrate increases cell proliferation (Yi et al., 2019; Yi et al., 2021b). Therefore, cell proliferation was quantified at 1, 3, 5, and 7 days. As proven by the previous studies, the enhanced cell proliferation of hADSCs was observed for the cells cultured on stiff nanofibers (SNE) after 5 days of culture (Figure 3C). Nevertheless, the cell spreading observed by SEM at 2 days exhibited no significant difference concerning the cell spreading area and adhered cell number (Figure 3D–F). This indicates that a short culture time was not sufficient to allow the different nanofibers to cause the obvious variation of cell behaviors. Moreover, live/dead assay was carried out to evaluate the cell viability of hADSCs on these PCL/Gel nanofibers. As shown in Figure 3G, a lot of live cells and a few dead cells were observed on both PCL/Gel nanofibers, revealing the good biocompatibility of the SVE-electrospun nanofibers.

The results, as mentioned above, revealed that the PCL/Gel nanofibers electrospun by SVE had weaker mechanical properties than those electrospun by SNE (Figure 2F). Usually, when cells contact the fiber surface, large protein complexes called focal adhesions (FAs) are formed to tether the cell cytoskeleton (e.g., integrins) to nanofibers. Then, the formed FAs repetitively tug onto the nanofibers to gauge their stiffness and remodel their structure by matrix assembling and matrix metalloproteinase secretion (Kennedy et al., 2017). Thus, a sufficient softness of scaffolds benefits cells to interact with the underlying matrix for remodeling (Meshel et al., 2005; Yi et al., 2019; Tang et al., 2021). To verify this, the expression of integrin-β1 in hADSCs was detected to observe the cell–fiber interactions after 3 days of cell culture. As shown in Figure 3H, both the PCL/Gel nanofibers were found to support cell growth and spreading along the direction of fiber orientation after the period of incubation. These observations indicate a good integration of hADSCs with PCL/Gel nanofibers.

## Conclusion

In this study, biocomposite PCL/Gel nanofibers were used for the comparative study of SNE and SVE. The numerical simulation revealed the expanded electric field distribution for high-throughput production of nanofibers in SVE. The results reported in physical properties demonstrated that although the type of spinneret (i.e., needle and spiral vane) has no significant impact on the surface chemistry, thermal stability, and composition of the obtained electrospun nanofibers, the PCL/Gel nanofibrous membranes produced by SVE exhibited higher air permeability (more wide distribution of fiber diameter) and better compliance (lower mechanical properties). Furthermore, the SVE-electrospun PCL/Gel nanofibers exhibited good biocompatibility for cell growth and cell–fiber interactions. This study provides a better understanding of the comparative study of SNE and SVE, offering essential knowledge in assisting the applications of SVE devices for the production of nanofibers to gain a larger market share, including tissue engineering, cosmetic product, and textile. In addition, although the results presented in this article were focused on PCL/Gel nanofibers, the observed performance variations of nanofibers can be speculated on other SVE-electrospun fibers, and the performed experimental scheme can also be a reference to the further comparative studies of other electrospinning setups.

## Data Availability

The original contributions presented in this study are included in the article/Supplementary Material, further inquiries can be directed to the corresponding authors.
